# COVID-19 contact tracing apps in Europe, technological feat or failure?

**DOI:** 10.1093/eurpub/ckac130.058

**Published:** 2022-10-25

**Authors:** A Santos, J Rachadell, R Vareda

**Affiliations:** 1 Public Health Unit, ACES Lisboa Ocidental e Oeiras, Oeiras, Portugal; 2 EUPHA-DH; 3 Public Health Unit, ACES Lisboa Norte, Lisbon, Portugal; 4 Public Health Unit, ACES Almada-Seixal, Almada, Portugal; 5 Institute for Evidence Based Health, Lisbon, Portugal

## Abstract

**Background:**

In the context of infectious disease control (IDC), public health services (PHS) have been under great pressure during the COVID-19 pandemic, especially with the burden of contact tracing. Most European Union countries have developed contact tracing apps for smartphones (CTA) with the goal of aiding PHS in IDC. CTAs generally use proximity data from mobile devices to determine a user's risk of exposure to SARS-CoV-2, thus providing testing and isolation recommendations. This review aimed to study the acceptance and adoption of CTAs in Europe.

**Methods:**

5 European countries were selected: Germany, Spain, France, Ireland, Italy. A literature review was carried out and official sources of information from each country were consulted in order to compare the adoption of each national CTA. Criterias included number of downloads, rating in the app stores, costo of development, proportion of positive tests registered. Potential factors influencing population adherence to CTAs were also investigated.

**Results:**

In proportion to their population, the number of downloads varied significantly in each country (18 % in Spain, 67% in France, 75% in Ireland). Except for Spain, all countries integrated additional functions into the CTA to increase its uptake, such as access to the vaccination certificate. App stores ratings ranged from 3.0 (Spain) to 3.9 (France and Ireland). The proportion of tests added in the apps varied significantly (1% in Italy and Spain, 4% in Ireland, 10% in France and 17% in Germany). Concerns that lead to the non-use of CTAs were related to data protection, smartphone battery drainage and app bugs.

**Conclusions:**

CTAs as a way to identify contacts from a positive case had a low impact in the analyzed countries, with low population adherence being an important factor. Adding more features within the apps, minimizing bugs, and increasing public confidence in data privacy seem essential to increase uptake in the future.

**Key messages:**

- Although conceptually attractive, CTAs have not proven to be effective in the context of the COVID-19 pandemic.
- There is a need for research and evaluation of the use of CTA, including cost effectiveness metrics, to inform future implementation similar technologies.

